# Electroacupuncture Preconditioning Reduces Oxidative Stress in the Acute Phase of Cerebral Ischemia-Reperfusion in Rats by Regulating Iron Metabolism Pathways

**DOI:** 10.1155/2021/3056963

**Published:** 2021-11-08

**Authors:** Runyu Liang, Qiang Tang, Wenjing Song, Mei Zhang, Lili Teng, Yuying Kang, Luwen Zhu

**Affiliations:** ^1^Heilongjiang University of Chinese Medicine, Harbin, Heilongjiang, China; ^2^Second Affiliated Hospital of Heilongjiang University of Chinese Medicine, Harbin, Heilongjiang, China; ^3^Fourth Affiliated Hospital of Heilongjiang University of Chinese Medicine, Harbin, Heilongjiang, China

## Abstract

**Background:**

Oxidative stress is an important mechanism of cerebral ischemia-reperfusion injury. Ferroptosis caused by iron overload after cerebral ischemia-reperfusion is considered a common cause of oxidative stress. Many recent studies have shown that electroacupuncture (EA) can regulate the expression of inflammatory factors, and the use of electroacupuncture preconditioning can produce a protective effect, which can reduce injury after cerebral ischemia and reperfusion. We aimed to assess whether EA could be used to reduce oxidative stress.

**Methods:**

The oxidative stress level of rats during the acute phase of cerebral ischemia and reperfusion was assessed with and without preconditioning with EA. Molecular biology methods were used to detect iron metabolism and oxidative stress-related proteins.

**Results:**

Rats that had EA preconditioning had lower infarct volumes than rats in the control group. Furthermore, western blot analysis showed that the expression of iron metabolism-related protein FPN-1 was higher in the intervention group than in the model group after reperfusion. In this regard, further investigation also demonstrated higher expression of glutathione and glutathione peroxidase-4, and lower reactive oxygen species values in the brain tissue of the EA group were compared with those of the control group rats.

**Conclusions:**

Electroacupuncture preconditioning can reduce oxidative stress after cerebral ischemia-reperfusion by regulating iron overload.

## 1. Introduction

Cerebral ischemia is a disease that endangers human health and quality of life and is the leading cause of death and disability worldwide [1]. After cerebral ischemia occurs, thrombolytic therapy is usually the first-choice treatment, and emboli can be surgically removed. However, when the blood supply is suddenly restored to ischemic and hypoxic brain tissue, it triggers oxidative stress, neuroinflammation, and a series of other complex cell cascade reactions [2]. This collection of conditions is known as ischemia-reperfusion injury and is considered a critical cause of disability after stroke treatment [3]. At present, reperfusion injuries are believed to be caused by immune cells and oxygen atoms in perfused blood [4, 5]. Moreover, reperfusion injury can affect the expression of hepcidin. As a regulator of iron balance, hepcidin can reduce the expression of ferroportin-1(FPN-1) so that iron ions can be retained in cells [6]. The iron ions in the cells cannot be transported in time, leaving the cells in a state of iron overload [7, 8]. In this state, the cell membrane is oxidized and broken by oxidative stress triggered by excessive free iron, resulting in ferroptosis [9, 10]. Nerve cells are greatly affected by damage caused by ferroptosis and other oxidative stress due to high oxygen consumption and difficulty in processing reactive oxygen species (ROS) [11]. Glutathione peroxidase-4 (GPX4) can deplete glutathione (GSH) to resist oxidative stress and is an important mechanism to resist oxidative stress in cells [12, 13]. Consequently, restoring the activity of GPX4 in cells and increasing GSH content will become a therapeutic strategy in combating oxidative stress damage.

Given the incidence and repercussions of oxidative stress after ischemia-reperfusion, it is vital to develop a treatment strategy that reduces oxidative stress. Administration of electroacupuncture (EA) stimulation before cerebral ischemia has been presented as an effective treatment strategy by several recent studies [14, 15]. EA not only induces ischemic tolerance but also inhibits the oxidative stress caused by reperfusion [16–19].

EA has been used as an effective and safe treatment for various diseases, especially brain diseases and their rehabilitation [20]. The effectiveness of electroacupuncture involves the acupuncture points, interval, intensity, and duration of intervention for stroke treatment [21]. Compared with other acupoints, we chose Baihui (GV20) and Zusanli (ST36). The literature has shown that electrical stimulation of Baihui can increase astrocytes and promote angiogenesis [22]. Electrical stimulation of Zusanli may cause increased cerebral blood flow in normal rats or those with ischemic stroke and can enhance the functional connection between the ipsilateral motor cortex and motor function-related brain areas (including the motor cortex, striatum, and sensory cortex) in focal ischemic rats [23, 24]. Furthermore, EA preconditioning can reduce the injury after cerebral ischemia-reperfusion and induce cerebral ischemic tolerance, and other effects have been confirmed [25, 26]. Therefore, we hypothesize that EA preconditioning can reduce the inflammatory response during cerebral ischemia-reperfusion and reduce the iron overload caused by oxidative stress.

Herein, we determined the effect of EA preconditioning on the acute phase of cerebral ischemia-reperfusion by assessing the levels of iron transport-related proteins and oxidative stress-related proteins during ischemia-reperfusion in a rat model.

## 2. Materials and Methods

### 2.1. Animals and Grouping

**Figure 1:**
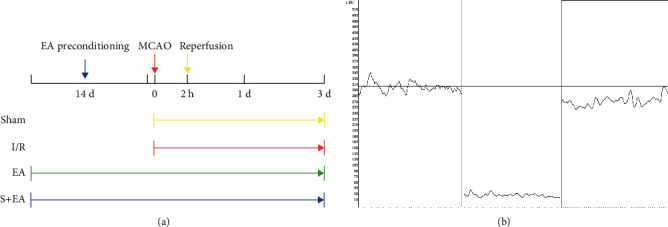
Schematic description of study design (a) and blood flow meter measurement icon (b). MCAO: middle cerebral artery occlusion; S: sham group; I/R: cerebral ischemia-reperfusion group; EA: electroacupuncture preconditioning group; and EA + S: electroacupuncture preconditioning and sham group.

Male Sprague Dawley rats aged 8–10 weeks (240 ± 20 g) were obtained from Liaoning Changsheng Biotechnology Co., Ltd. (SCXK 2015-0001). Rats were housed in a ventilated room with free access to food and water and maintained at a temperature of 24 ± 2°C, with a humidity of 60 ± 5%, and a day/night cycle was created by alternating between bright and dark lighting every 12 h. The rats were divided into 4 groups of 12 rats, allocated by a random number generator, and reared independently. The rats were divided into four groups: the sham group (sham) (*n* = 12), cerebral ischemia-reperfusion group (I/R), electroacupuncture preconditioning group (EA), and electroacupuncture preconditioning and sham group (EA + S). Each group was subdivided for sacrifice at either 1 day (*n* = 6) or 3 days (*n* = 6) after reperfusion. The experimental protocol is shown in Figure 1(a). The experiment was approved by the Animal Care and Use Committee at the Heilongjiang University of Chinese Medicine, and all animals were euthanized in accordance with the National Institutes of Health Guidelines.

### 2.2. Electroacupuncture Preconditioning Protocol

EA preconditioning was performed 14 days before Middle Cerebral Artery Occlusion (MCAO) modeling. First, the rat was fixed on a special fixator, and the Baihui point (GV20) was punctured to a depth of 4 mm and Zusanli point (ST36) to a depth of 8 mm and an angle of 15°, with a 0.13 × 0.25 mm acupuncture needle (Hwato, Suzhou Medical Appliance Factory, China). The EA treatment instrument (G6805-2A, Shanghai Huayi Group, China) was used to simultaneously stimulate the acupoints. The positive pole of the instrument was connected to Baihui (GV20), and the negative pole was connected to Zusanli (ST36) using the density wave, frequency 2/15 Hz, stimulation intensity 1 mA, 30 min per day, 6 days a week, and total two weeks. The sham and I/R groups did not undergo EA preconditioning, but rats were handled daily, similar to those experiencing EA.

### 2.3. I/R Model

The I/R model was established 24 h after the two-week EA preconditioning, as described previously [27, 28]. Briefly, the rats were placed on an animal anesthesia machine (RWD510, RWD Life Science Inc., China) and anesthetized with 5% isoflurane inhalation. Next, a small opening was made in the common carotid artery on the left, and a wire thread embolus (A4-263450, Beijing Cinotech Co., Ltd., China) with a tip coated with silicone was inserted approximately 18–20 mm and reached the middle cerebral artery; this technique effectively occluded cerebral blood flow. After 2 h of ischemia, the thread embolus was pulled out approximately 10 mm to achieve reperfusion. A laser Doppler flow meter (PeriFlux 5100 Laser Doppler, PERIMED Inc., Sweden) was used to record the situation before ischemia, after ischemia, and after reperfusion to determine the success of ischemia and reperfusion (Figure 1(b)). In the sham groups, except for inserting the thread embolus in the left common carotid artery, the rest of the operations were the same as those in the I/R group. Rats that failed or died were excluded and replaced by rats that met the criteria.

### 2.4. 2, 3, 5-Triphenyltetrazolium Chloride (TTC) Staining

TTC staining is used to verify the success of the stroke model and determine infarct volume. One day after reperfusion, each rat was deeply anesthetized, sacrificed, and dissected on ice. Brain tissue was removed and placed in a freezer at −20°C for 30 min. The frozen brain tissue was separated from the cerebellum and dissected into 2 mm sections from the optic chiasm. Next, brain tissue was placed in a 2% TTC solution (G3005, Solarbio Inc., China) in a 37°C incubator for 10–15 minutes, and samples were observed and removed when adequate tissue color change was achieved. The successfully stained brain tissue section showed infarcts in white and normal brain tissue in red and was fixed in 4% paraformaldehyde once the desired coloration was achieved. A high-definition camera was used for imaging and calculation of the infarct area, measured by Image-Pro Plus 6.0 software (Media Cybernetics Inc., America). The infarct area was determined as the infarct volume÷ total brain tissue volume × 100% and expressed as a percentage.

### 2.5. Hematoxylin-Eosin (HE) Staining

The rat brain, which was perfused with 9% physiological saline and 4% paraformaldehyde placed in the tissue fixative, was used for pathological observation using the hematoxylin and eosin staining method. First, the dehydrated brain tissue was sliced and embedded in paraffin. After gradient xylene and alcohol treatment, the brain tissue was immersed in hematoxylin and eosin staining solutions. The successfully stained tissue sections were fixed for observation and microscopy. The main observation locations are the cerebral cortex and ischemic penumbra.

### 2.6. Western Blot Analysis

The rat infarcted side cortex or ischemic penumbra brain tissue was treated with RIPA Lysis Buffer (P0013B-RIPA, Beyotime, China) for grinding to extract total protein. After measuring the protein, samples were prepared and run by 10% sodium dodecyl sulfate-polyacrylamide gel electrophoresis and then transferred onto a polyvinylidene difluoride membrane. The imprinted polyvinylidene fluoride (PVDF) membranes were blocked in a sealed bag at room temperature for 2 h with skimmed milk powder dissolved in Tris-buffered saline with Tween solution (50 mmol/L Tris-HCl, pH 8.0, 150 mmol/L NaCl, and 0.1% Tween-20). The membranes were then blocked with FPN-1 primary antibodies (1 *μ*g/mL, 26601-1-AP, Proteintech, China) for 1.5 h at room temperature (25°C ± 1°C), according to the manufacturer's instructions. Finally, the membranes were incubated with HRP-labeled goat anti-rabbit immunoglobin G antibody for 2 h in a shaker. *β*-Actin polyclonal antibody (0.5 *μ*g/mL, 20536-1-AP, Proteintech, China) was used as an internal reference. Proteins were detected with enhanced ECL chemiluminescence substrate, and images were taken to measure quantitative expression using Gel-Pro-Analyzer 4.0 (Media Cybernetics, USA) and Image J (National Institutes of Health, America).

### 2.7. Enzyme Linked Immunosorbent Assay (ELISA)

ELISA was used to detect the protein content. According to the manufacturer's instructions (MEIMIANbio, China), PBS was used as a protein diluent. The sample (10 *μ*l) was added to a microcuvette coated with hepcidin, GSH, GPX4, and ROS antibodies (MEIMIANbio, China), followed by 50 *μ*l of sample diluent and 100 *μ*l of enzyme-labeled reagent. The microcuvettes were then incubated for 1 h at 37°C. After the initial incubation, the cuvettes were washed five times using the washing solution provided in the kit. The A and B reaction solutions were added, and the cuvettes were incubated for a further 15 min in the dark. Finally, 50 *μ*l of stop solution was added to stop the reaction. The protein content was measured using a microplate reader with a 450 nm filter (Multiskan FC, Thermo Fisher, USA).

### 2.8. Statistical Analysis

All experimental data were analyzed using SPSS 26.0 (IBM Inc., America) and displayed in the form of mean ± standard deviation. The quantitative data for comparing the two groups were tested using Student's *t*-test, while multiple groups of data considering single-factor changes were statistically analyzed using analysis of variance, and multiple post hoc comparisons were used to pass the LSD test. Differences were considered statistically significant when *P* was <0.05.

## 3. Results

### 3.1. TTC Staining

**Figure 2:**
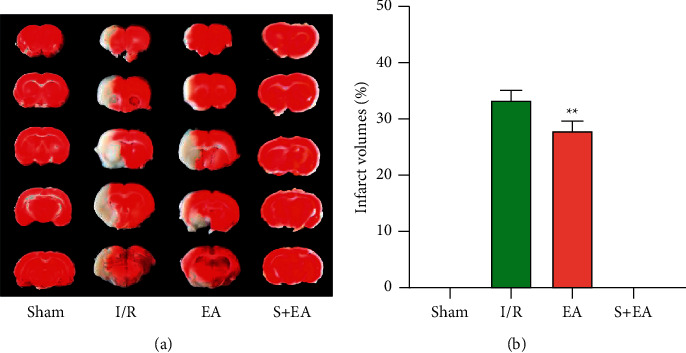
EA preconditioning exerted a neuroprotective role in cerebral ischemia/reperfusion rats. (a) Sequential cerebral slices of tissue from single rats in the group which was sacrificed 1 day were stained with 2% TTC solution. (b) Cerebral infarction volume measurement through Image-Pro Plus (^*∗∗*^*P* < 0.01 vs. I/R group). S: sham group; I/R: cerebral ischemia-reperfusion group; EA: electroacupuncture preconditioning group; and EA + S: electroacupuncture preconditioning and sham group.

TTC staining showed that the infarct volume of rats pretreated with electroacupuncture for two weeks was smaller than that of the I/R group after 1 day of cerebral ischemia and reperfusion. The calculated infarct volume was significantly smaller in the EA group (28.14% ± 1.46%) than in the I/R group (33.48% ± 1.44%) (*P* < 0.01) (Figure 2).

### 3.2. HE Staining

**Figure 3:**
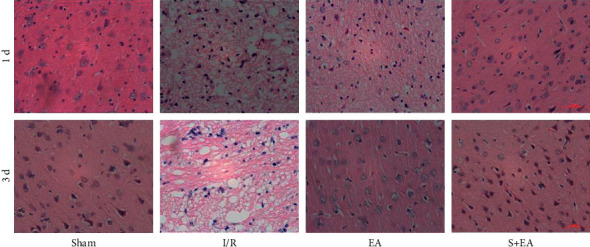
EA preconditioning can reduce acute pathological damage after cerebral ischemia and reperfusion. 1 d: 1 day after reperfusion; 3 d: 3 days after reperfusion; S: sham group; I/R: cerebral ischemia-reperfusion group; EA: electroacupuncture preconditioning group; and EA + S: electroacupuncture preconditioning and sham group.

HE staining more effectively demonstrated the changes in brain tissue after cerebral ischemia and reperfusion. The cells in the sham and EA + S groups were arranged neatly, with clear and complete levels. The brain tissue of the I/R group was loose and edematous, showing vacuolar changes, and the nucleus was pyknotic. The degree of brain edema and vacuole changes in the EA group was lower than that in the I/R group, although the degree of damage was different. It should be noted that brain tissue damage increases with time after reperfusion, but EA preconditioning appeared to reduce this damage (Figure 3).

### 3.3. Western Blot Analysis

**Figure 4:**
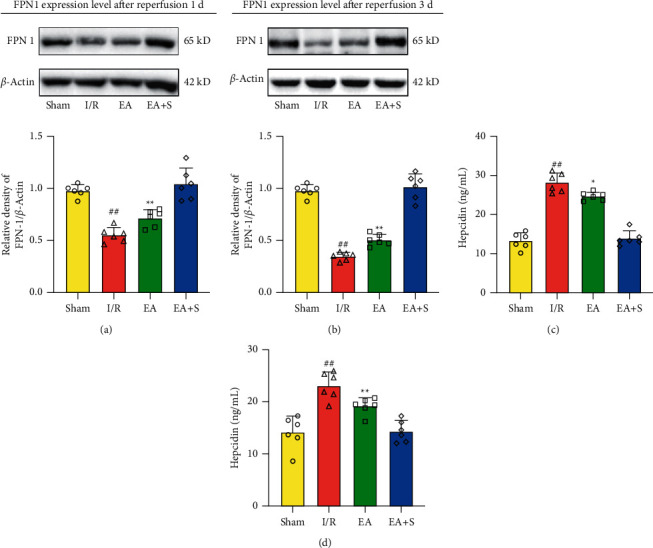
Effect of EA preconditioning on iron transport-related protein in the acute phase of cerebral ischemia and reperfusion. (a) The effect of EA preconditioning on the expression of FPN-1 in rats sacrificed 1 day after reperfusion. (b) The effect of EA preconditioning on the expression of FPN-1 in rats sacrificed 3 days after reperfusion. (c) The effect of EA preconditioning on the content of hepcidin in brain tissues of rats sacrificed 1 day after reperfusion. (d) The effect of EA preconditioning on the content of hepcidin in brain tissues of rats sacrificed 3 days after reperfusion (^*∗*^*P* < 0.05 vs. I/R group; ^*∗∗*^*P* < 0.01 vs. I/R group; and ^##^*P* < 0.01 vs. sham group and EA + S group). S: sham group; I/R: cerebral ischemia-reperfusion group; EA: electroacupuncture preconditioning group; and EA + S: electroacupuncture preconditioning and sham group.

The FPN-1 level in the EA group was significantly higher in the brain tissue of the infarct area than in the I/R group (*P* < 0.01), but it was still significantly lower than in the sham and EA + S groups. This phenomenon was still present after 3 days of reperfusion (*P* < 0.01) (Figures 4(a) and 4(b)). Additionally, the hepcidin content in the brain tissue was lower than that in the I/R group at both time points (*P* < 0.01) (Figures 4(c) and 4(d)).

### 3.4. ELISA

**Figure 5:**
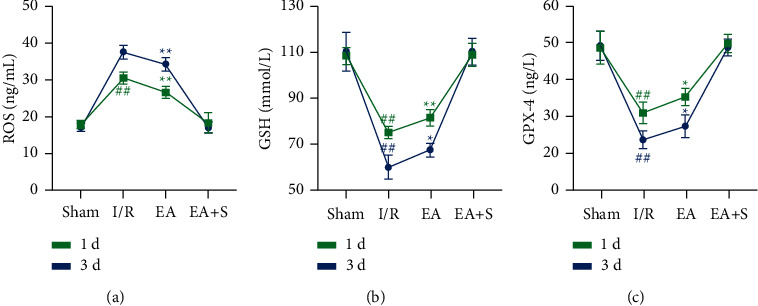
Effect of EA preconditioning on the content of oxidative stress-related proteins. (a) EA preconditioning can reduce the content of ROS in brain tissues in the acute phase after reperfusion. (b) EA preconditioning can reduce the content of GSH in brain tissues in the acute phase after reperfusion. (c) EA preconditioning can reduce the content of GPX4 in brain tissues in the acute phase after reperfusion (^*∗*^*P* < 0.05 vs. I/R group; ^*∗∗*^*P* < 0.01 vs. I/R group; and ^##^*P* < 0.01 vs. sham group and EA + S group). S: sham group; I/R: cerebral ischemia-reperfusion group; EA: electroacupuncture preconditioning group; and EA + S: electroacupuncture preconditioning and sham group.

Compared with the I/R group, the EA group had a lower oxidative stress level, as measured by ROS, at 1 and 3 days after reperfusion (*P* < 0.01) (Figure 5(a)). Furthermore, concentrations of GSH and GPX4, which can protect cells by inhibiting oxidative stress and ferroptosis caused by iron overload, were significantly higher in the EA group than in the I/R group, at both time points (*P* < 0.05) (Figures 5(b) and 5(c)).

## 4. Discussion

Although studies pertaining to the development and progress of stroke have provided a clearer understanding of ischemic diseases, we do not have a perfect treatment plan yet. Even when strokes are detected and treated as early as possible, thrombolysis is still a mode of treatment with certain risks [29]. With prolonged ischemia and hypoxia, the infarction gradually expands. Reversing the injury as soon as possible is preferable, but if a patient has already experienced prolonged ischemia, this can cause reperfusion injury with serious consequences [30].

As a conventional measure, therapy to restore perfusion has more benefits than risks and can improve survival rates. It should be noted that, unlike permanent ischemia, reperfusion injury changes the physiological state after ischemia, in a process more related to inflammation [31]. Not only can ischemic changes cause the expression of various inflammatory factors but also the immune cells in the perfused blood cannot be ignored [32] and can trigger severe inflammation, which is an important mechanism leading to iron overload and ferroptosis in cells [33].

Acupuncture has emerged as a supplementary (available here) and alternative treatment for stroke. The mechanisms of action have been investigated in several studies, which have shown a reduction in reperfusion inflammatory reactions and ferroptosis [34, 35]. EA has the advantages of continuous acupuncture, relatively fixed parameters, and reduced manpower and has been suggested as a strategy to combat damage caused by cerebral ischemia [21]. Baihui (GV20) is often selected as a treatment for cerebral ischemia because it has many functions in traditional Chinese medicine, such as dredge collateral, regulation of qi flow, restoration of consciousness, and benefitting resuscitation [36]. Many studies have demonstrated the neuroprotective effect of treating the Baihui (GV20) point with electroacupuncture [37, 38]. Additionally, Zusanli (ST36) also has an effect that makes the body strong in traditional Chinese medicine; therefore, it is widely used in clinical treatment [36]. Previous studies on the effect of Zusanli in stroke diseases have shown that it enhances limb movement [39]. In summary, we chose the Baihui (GV20) point and Zusanli (ST36) point as the EA preconditioning protocol because they are often used in clinical treatment and have good curative effects. We selected the electrical component of the treatment (stimulus and frequency) based on previous research [40, 41].

TTC staining showed a smaller infarct volume in the EA group than in the I/R group. This finding supported the pathological staining results, demonstrating less damage in the EA group than in the I/R group. We investigated the mechanism of this reduction by analyzing the levels of hepcidin and FPN-1 within the cortex tissue. Hepcidin can be activated by the HAMP gene, which is upregulated by the IL-6/JAK/STAT3 signaling pathway [42]. FPN-1, an iron-transporting protein that can transport iron ions from cells to plasma, is downregulated by hepcidin [43]. The results showed that EA preconditioning could reduce the increase of hepcidin caused by cerebral ischemia-reperfusion within 3 days and restore the expression level of FPN-1. The reduction in ROS levels also supports this conclusion as it indicates the absence of excess intracellular iron in the tissue from the EA group rats.

In addition, we assessed the levels of GSH and GPX4 in the brain tissue. GSH and GPX4 can antagonize the Fenton reaction, produced by a large amount of intracellular iron, and protect the cell membrane from oxidative damage [44]. This indicates that EA preconditioning protects against the consumption of GSH and GPX4. Higher levels of GSH and GPX4 also indicate reduced oxidative stress levels in the brain tissue and low occurrence of ferroptosis.

Consequently, we believe that EA can reduce the consumption of GSH and keep GPX4 active to resist lipid peroxidation. Simultaneously, EA can reduce the generation of ROS and protect cells. Taken together, we believe that EA can exert neuroprotective effects after cerebral ischemia and reperfusion. The effect can be sustained, at least in the acute to subacute phase.

Although the experimental results reveal the advantages of EA preconditioning to reduce the injury after cerebral ischemia and reperfusion and exert neuroprotective effects, this experiment also has certain limitations. We demonstrated that EA preconditioning could reduce injury after ischemia-reperfusion; however, the research is based on animal experiments. Even though we used the left hemisphere of the brain as the object of ischemia, as suggested by a previous review [28], the experiment using only adult male rats does not cover all types of disease. Therefore, future directions will include exploring the limitations of this research and aim to conduct more in-depth research into the mechanisms of action.

As most previous studies focused on the mechanism of electroacupuncture preconditioning, the duration of its effect remains unclear; therefore, in the future, more attention should be paid to calculating the protection time of electroacupuncture preconditioning, and the mechanism should be analyzed to optimize electroacupuncture points, parameters, and intervention cycles, etc., to provide a better reference for clinical services.

## 5. Conclusions

We found that electroacupuncture preconditioning can protect nerve cells from oxidative stress damage after cerebral ischemia and reperfusion by regulating iron transport-related proteins, which is consistent with previous research and our hypothesis. However, the mechanism of ferroptosis and how to prevent it remains a problem requiring an urgent solution.

## Data Availability

The experimental data of this study are stored in the FAIRDOMHub database [45]: https://fairdomhub.org/projects/251/.

## Supplementary Materials

Supplementary Materials The ARRIVE Compliance Questionnaire, etc. supporting this study are stored in the FAIRDOMHub database as supplementary files.
